# Experimental Infection of Macaques with a Wild Water Bird-Derived Highly Pathogenic Avian Influenza Virus (H5N1)

**DOI:** 10.1371/journal.pone.0083551

**Published:** 2013-12-18

**Authors:** Tomoko Fujiyuki, Misako Yoneda, Fumihiko Yasui, Takeshi Kuraishi, Shosaku Hattori, Hyun-jeong Kwon, Keisuke Munekata, Yuri Kiso, Hiroshi Kida, Michinori Kohara, Chieko Kai

**Affiliations:** 1 Laboratory Animal Research Center, The Institute of Medical Science, The University of Tokyo, Tokyo, Japan; 2 Department of Microbiology and Cell Biology, Tokyo Metropolitan Institute of Medical Science, Tokyo, Japan; 3 Amami Laboratory of Injurious Animals, The Institute of Medical Science, The University of Tokyo, Kagoshima, Japan; 4 Graduate School of Veterinary Medicine, Hokkaido University, Hokkaido, Japan; Institut Pasteur, France

## Abstract

Highly pathogenic avian influenza virus (HPAIV) continues to threaten human health. Non-human primate infection models of human influenza are desired. To establish an animal infection model with more natural transmission and to determine the pathogenicity of HPAIV isolated from a wild water bird in primates, we administered a Japanese isolate of HPAIV (A/whooper swan/Hokkaido/1/2008, H5N1 clade 2.3.2.1) to rhesus and cynomolgus monkeys, in droplet form, via the intratracheal route. Infection of the lower and upper respiratory tracts and viral shedding were observed in both macaques. Inoculation of rhesus monkeys with higher doses of the isolate resulted in stronger clinical symptoms of influenza. Our results demonstrate that HPAIV isolated from a water bird in Japan is pathogenic in monkeys by experimental inoculation, and provide a new method for HPAIV infection of non-human primate hosts, a good animal model for investigation of HPAIV pathogenicity.

## Introduction

Highly pathogenic avian influenza virus (HPAIV) infection in humans was first recognized in 1997 [1,2]. Since then, HPAIV has continuously been isolated globally from wild birds [3] and studies aimed at developing a means of defense against HPAIV are in progress. 

 Non-human primate models of infection are urgently required for investigation of the pathogenesis of HPAIV in humans and for development of new vaccines or drugs against HPAIV. However, there have only been a few studies of HPAIV infection in rhesus macaques [4,5]. In addition, the pathogenesis of HPAIV has been studied using clinically isolated viruses derived from human patients in a limited number of countries. Therefore, there is little knowledge about how HPAIV is transmitted from wild water birds to humans. Influenza virus strain A/whooper swan/Hokkaido/1/2008 (H5N1 clade 2.3.2.1) was isolated from a dead wild water bird in Japan. Its high pathogenicity was confirmed using chickens [6]. In the present study, we examined pathogenicity of HPAIV derived from a wild water bird in non-human primate. 

 Furthermore, the route of inoculation needs consideration. Theoretically, there are three modes of transmission of influenza virus: airborne (aerosol), droplet, and contact [7,8]. In previous macaque infection models, a large amount of liquid containing influenza virus was poured intratracheally into the lungs, which cannot occur during natural transmission [4,5,9-12]. Droplet exposure would more closely mimic natural transmission between animals. Thus, we investigated whether droplet exposure of macaques to this Japanese HPAIV strain could induce influenza symptoms using two different macaque species, rhesus and cynomolgus monkeys, with the aim of understanding the virulence of this strain in non-human primates, the efficacy of droplet exposure for infection, and common or different properties of macaque species in terms of HPAIV pathogenicity.

## Materials and Methods

### Ethics statement

All animal experiments followed the Regulations for Animal Care and Use of the University of Tokyo and were approved by the Animal Experiment Committee at the Institute of Medical Science at The University of Tokyo (approval number: A10-54). All surgery was performed under anesthesia with a ketamine-xylazine combination, and all efforts were made to minimize animal suffering. At the end of the experimental period, the monkeys were euthanized by anesthesia with a ketamine-xylazine combination followed by exsanguination. Monkeys were kept in an enriched environment, which was achieved as follows: (1) monkeys were given pellet, fruits, and sweet potato once a day in the morning, and they had free access to food and water; (2) individual cages had bars that the monkeys could use for climbing up and down; (3) the monkeys could see and hear each other. Amplification of the virus in embryonating chicken eggs was conducted in accordance with guidelines of the Institutional Animal Care and Use Committee of Tokyo Metropolitan Institute of Medical Science. 

### Virus

A/whooper swan/Hokkaido/1/2008 (H5N1 clade 2.3.2.1) was originally isolated from a dead wild water bird in Japan [6]. The virus was amplified in embryonating chicken eggs [6]. Virus titer was measured by plaque assay using Madin-Darby canine kidney (MDCK) cells. Ten-fold dilutions of virus were inoculated onto confluent MDCK cells and incubated at 37 °C for 1 h. Unbound viruses were removed, and the cells were then overlaid with MEM containing 0.8% agarose (Sigma, type II), 1% BSA (Sigma) and 10 μg/ml acetyl trypsin (Sigma). After 72 h of incubation at 37 °C, cells were stained with 1% crystal violet. All infectious work with the virus was performed in biosafety level 3 laboratories in the Institute of Medical Science, The University of Tokyo and in Tokyo Metropolitan Institute of Medical Science. 

### Monkeys

Eight rhesus monkeys (*Macaca mulatta*, 1 year old, weighing 1.4 to 2.0 kg) and six cynomolgus monkeys (*Macaca fascicularis*, 5-6 years old, weighing 2.6 to 3.8 kg) were maintained in the Amami Laboratory of Injurious Animals of the Institute of Medical Science, The University of Tokyo. Body temperatures and activity of the monkeys, were monitored using a telemetry system (Data Sciences International, Saint Paul, MN). Surgeries to implant the telemetry transmitter (TA10CTA-D70) were performed under anesthesia with a ketamine-xylazine combination. Monkeys were individually housed in cages (L: 560 mm, H: 767 mm, W: 700 mm). 

### Virus inoculation

Monkeys were anesthetized with a ketamine-xylazine combination. Different combinations of three different routes were used to inoculate animals as shown in Table S1. Briefly, virus solution (3 × 10^6^ PFU, 1 × 10^7^ PFU, or 1 ×10^8^ PFU) was inoculated into monkeys intratracheally and intranasally by droplet (four cynomolgus monkeys 23−25 and 44, seven rhesus monkeys 27−33) or by catheter (two cynomolgus monkeys 34 and 59, and one rhesus monkey 26), and orally by pipette. Intratracheal, nasal, and oral inoculation was performed twice in the same animal. For droplet exposure, a custom-made MicroSprayer (IA-1B, PennCentury, Wyndmoor, PA) 33 cm long and with a diameter of 1.5 mm was connected to a 1-ml syringe. The device is based on a 19-gauge, stainless steel tube and delivers a plume of aerosol (mass median diameter of 25−30 μm, http://www.penncentury.com/products/IA_1B.php). It has been used to administer droplets to animal lungs [13,14]. In addition, we obtained preliminary data using mice that suggested that the MicroSprayer inoculation method can cause a broader distribution of inoculum in the lung than a conventional inoculation method (Figure S1). In our monkey experiment, the MicroSprayer was inserted into the trachea to a depth of approximately 5 cm from the epiglottis with the aid of a laryngoscope, and then virus solution was atomized. 

### Pathological examination

Animals were observed daily for clinical signs. At 1−7 days post-inoculation (dpi) for cynomolgus monkeys, or 2, 4, 6, and 7 dpi for rhesus monkeys, body weight and respiratory rates were recorded and chest radiographic photographs were taken under anesthesia. Blood, and oral and nasal swabs were also simultaneously collected and then suspended in 0.75 ml of TRIzol reagent (Invitrogen, Carlsbad, CA) for RNA extraction or in 1 ml of Hanks’ medium containing antibiotics. Virus titration in swab samples was performed using MDCK cells and the titer was described as TCID_50_/ml. At 7 dpi, animals were euthanized and clinical specimens from nasal mucosa, nasal septum, trachea, bronchi, lungs, hilar lymph nodes, heart, liver, spleen, kidney, jejunum, rectum, brain and mesenteric lymph nodes were collected. Animal tissues were fixed in 10% phosphate-buffered formalin, and embedded in paraffin and sections were stained with hematoxylin and eosin. The severity of inflammation was scored as follows: 0, none; 1, mild (area of inflammation in the section was ≤ 1/3 of the observed section); 2, intermediate (area of inflammation in the section was between 1/3 and 2/3 of the observed section); 3, severe (the area of inflammation in the section was ≥ 2/3 of the observed section).

### One step RT-PCR

Total RNA was extracted from each frozen macaque organ using TRIzol reagent. One-step RT-PCR was performed similarly to a previous report [15]. Here, 100 ng of total RNA was used with SuperScript One-Step RT-PCR with Platinum Taq (Invitrogen) and specific primers for the matrix (M) gene of the virus (forward; 5’- CTTCTAACCGAGGTCGAAACGTA - 3’, reverse; 5’- CTGGACAAAACGTCTACGCTGC - 3’). RT-PCR conditions used were: 55°C for 30 min + 94°C for 2 min + (94°C for 15 s + 55°C for 30 s + 68°C for 30 s) × 45 cycles. The presence of virus RNA was determined by DNA amplification of the expected length. Swab samples were similarly analyzed. 

### Quantitative RT-PCR (qRT-PCR)

One microgram of total RNA was reverse transcribed with PrimeScript (Takara, Japan) using a random hexamer. PCR was then performed with Rotor-Gene Q system (Qiagen, Germany) and Thunderbird SYBR qPCR mix (Toyobo, Japan) using M-specific primers as described above in a total of 20 µl. PCR conditions used were: (95°C for 15 s + 55°C for 15 s + 72°C for 45 s) x 40 cycles. A cDNA clone of M gene of the same viral strain was serially diluted and used as a standard. 

### Hematology

The total numbers of white blood cells, platelets and red blood cells in EDTA blood were determined using a hematology analyzer (Sysmex, Japan). The proportion of lymphocytes was determined by flow cytometry analysis. Whole blood was incubated at room temperature for 30 min in the dark with CD4 (OKT4 conjugated with APC, eBioscience), CD8 (RPA-T8 conjugated with FITC, BioLegend), CD56 (B159 conjugated with PE, BD Bioscience), or CD20 (L27 conjugated with PE, BD Bioscience) monoclonal antibodies. Red blood cells were lysed using an ammonium chloride solution and the resultant leukocytes were washed twice with PBS, and then fixed with formalin. Cells were gated based on their forward and side scatter properties.

### Data analysis

A Chi-square analysis or Wilcoxon rank sum test was used for analyzing virus amount and distribution (cut off=10^3^ copies). Viral shedding was analyzed using one-way repeated measures ANOVA. Statistical significance was defined as *p*<0.05. All statistics were performed using JMP software (JMP Pro 10.0.2, SAS Institute Inc., Cary, NC). Telemetry data was converted to the area of under the curve using Photoshop CS6 (Adobe Systems, CA). The AUC value for each day was compared to that on the pre-inoculation day. 

## Results

### HPAIV infection in two macaques after droplet exposure

**Figure 1:**
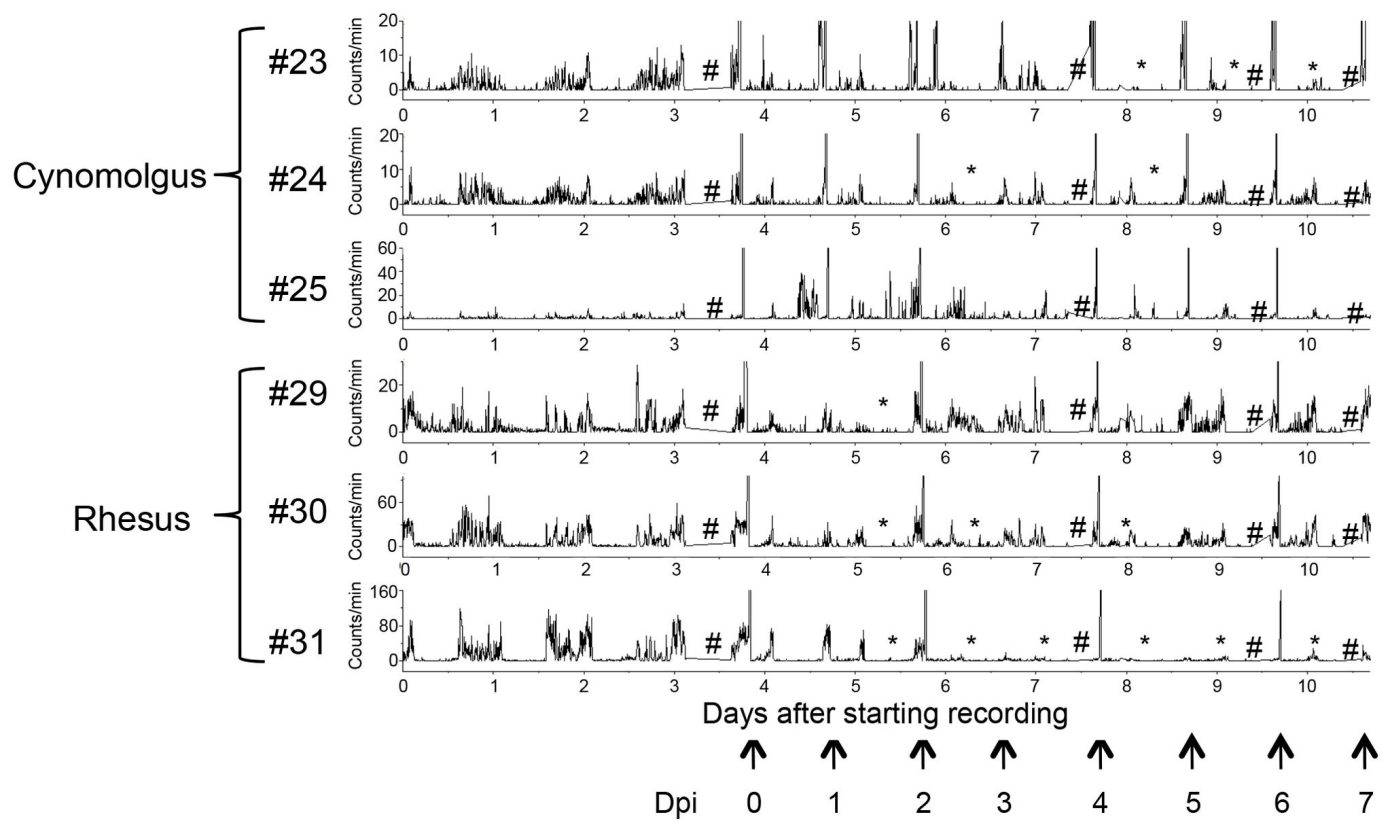
Activity of cynomolgus and rhesus monkeys challenged with HPAIV. The activity of each monkey challenged with 3 × 10^6^ PFU of HPAIV was monitored by a telemetry system. Amplitude indicates activity of an animal. *Period with decreased activity; AUC was decreased to ≤ 50% of the pre-inoculation level. #Data not obtained. The arrow indicates the time of anesthesia for sampling. Hyperthermia was caused by anesthesia.

**Figure 2:**
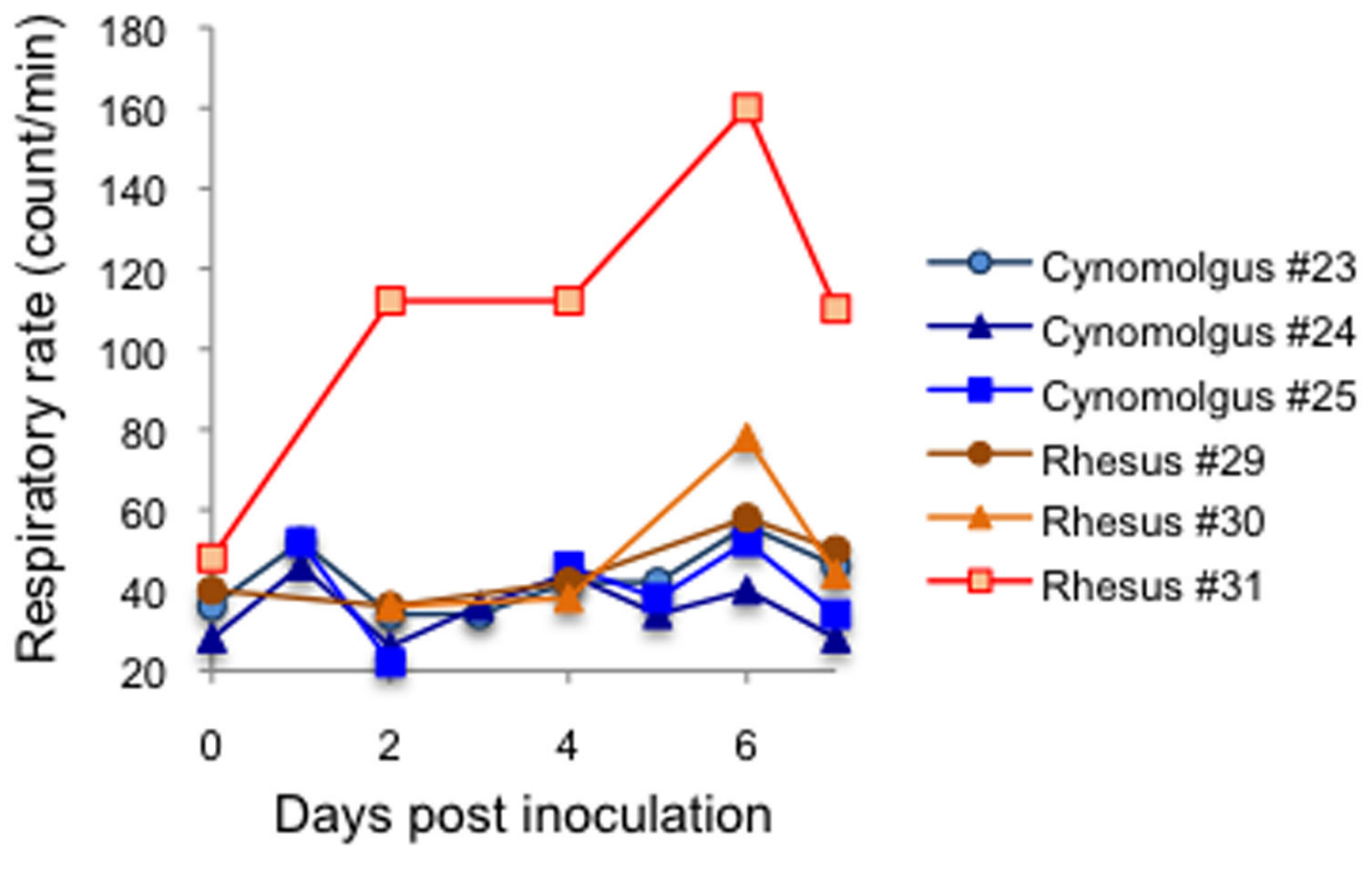
Changes in respiratory rate in HPAIV-challenged monkeys. The respiratory rate was measured soon after anesthesia, daily (cynomolgus monkeys) or at 0, 2, 4, 6, and 7 dpi (rhesus monkeys).

To examine whether a Japanese HPAIV isolate from a wild water bird [A/whooper swan/Hokkaido/1/2008 (H5N1)] is pathogenic to primates and which monkey species are highly susceptible to HPAIV infection, rhesus and cynomolgus monkeys were inoculated with 3 × 10^6^ PFU of the virus via multiple routes, including intratracheal, intranasal and oral routes. For intratracheal and intranasal inoculations, we used the MicroSprayer to mimic droplet exposure. Challenged animals showed mild clinical signs. The activity of most monkeys was decreased after inoculation (Figure 1). The decreased activity was maintained until necropsy in some of the monkeys (cynomolgus #23, rhesus #31). Tachypnea was obvious in one of the rhesus monkeys (Figure 2; 31). Body weight was not significantly changed (data not shown).

**Figure 3:**
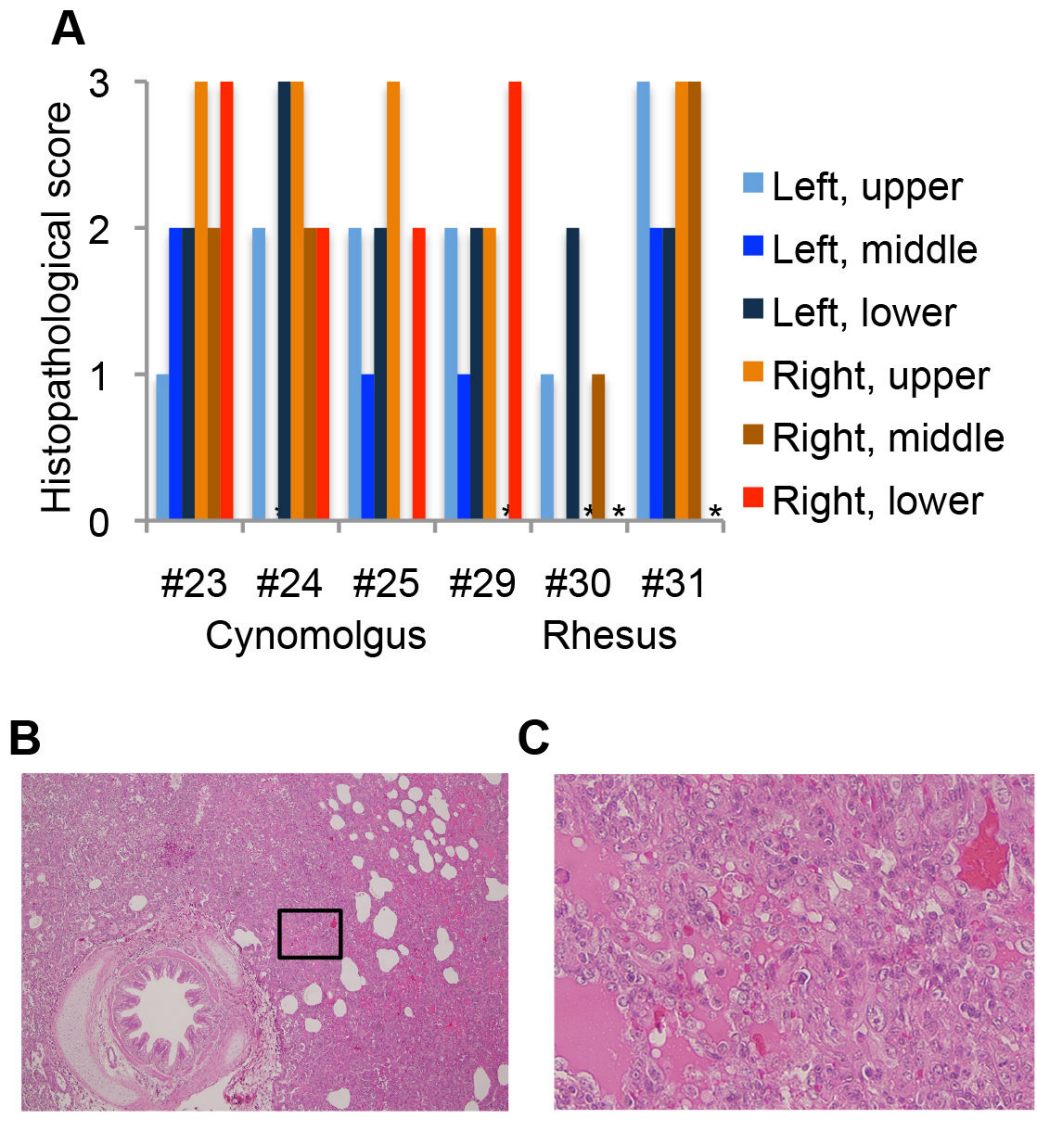
Histopathology of the lungs of cynomolgus and rhesus macaques challenged with HPAIV. (A) Histopathology of the lung lobes was scored as: 0, none; 1, mild (area of inflammation was ≤1/3 of the observed section); 2, intermediate (area of inflammation was between 1/3 and 2/3 of the observed section); 3, severe (area of inflammation was ≥2/3 of the observed section). * Not analyzed. (B) Hematoxylin and eosin stains of the right upper lobe of the lungs of rhesus monkey 31 (×40). (C) Inset of (B) is magnified (×400).

 The monkeys were euthanized at 7 dpi and their organs were analyzed. Histopathological analysis of the lungs of the challenged monkeys revealed signs of interstitial pneumonia such as infiltration of immune cells, mainly lymphocytes, and alveolar edema (Figure 3). Rhesus monkey 31 showed the most severe lesions in the lungs, with histopathological scores of the tested lung lobes higher than 2. This is consistent with the observation that rhesus monkey 31 showed severe tachypnea (Figure 2). 

**Figure 4:**
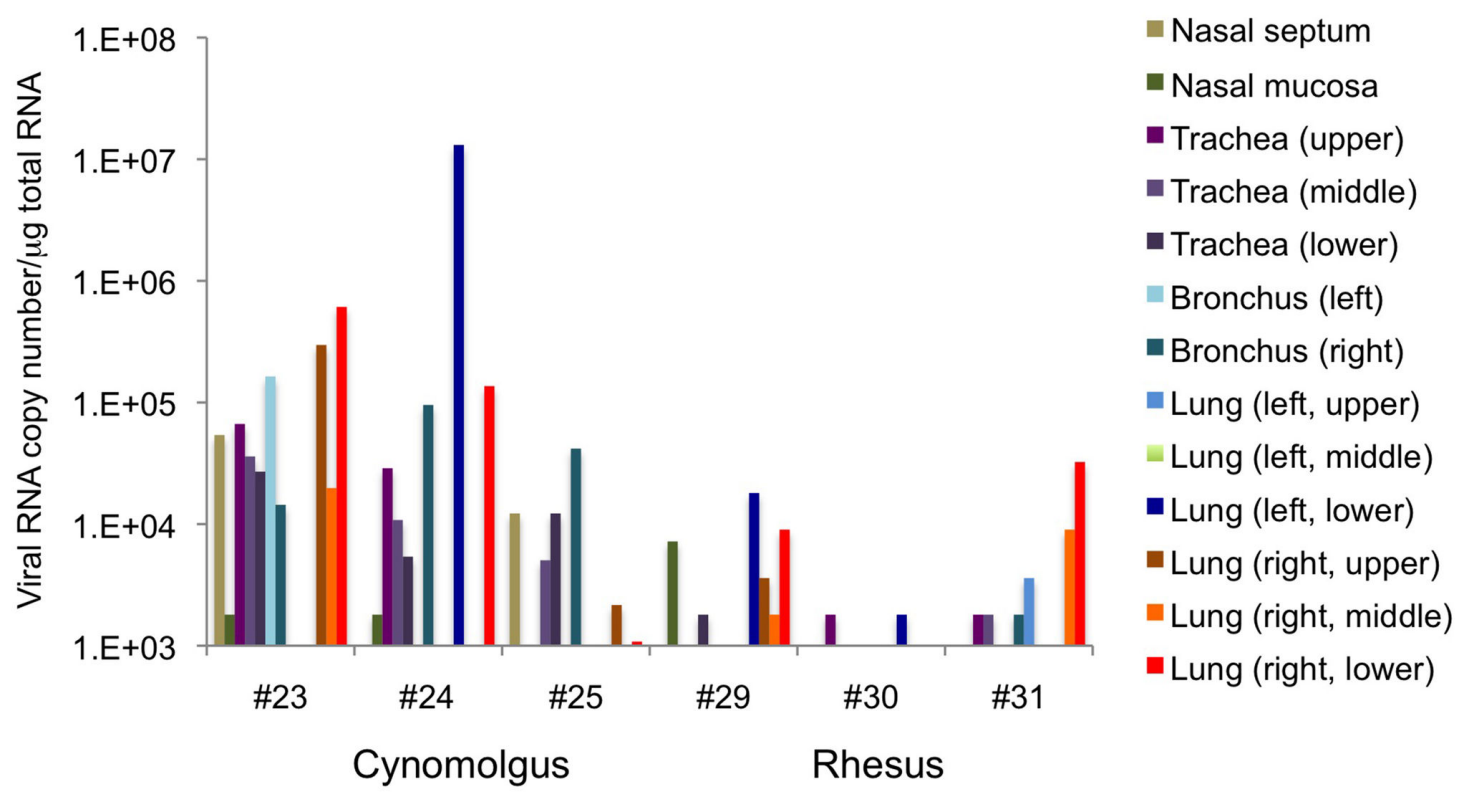
The amount of viral RNA in the respiratory tract between cynomolgus and rhesus macaques. Viral RNA was quantified by qRT-PCR. The log value of the relative viral RNA content is indicated.

 RT-PCR analysis showed that viral RNA was present in the respiratory tracts of all monkeys (Figure 4), although the amount detected and the area infected varied among individuals. This result suggests that HPAIV successfully infected the respiratory tract and caused pneumonia in both macaque species, thus, the Japanese isolate from a wild water bird is useful for investigation of HPAIV pathogenicity in macaques. The amount and distribution of the viral infection in the respiratory tract was higher (p=0.001, Wilcoxon rank sum test) and broader (p=0.02, Chi Square Approximation) in cynomolgus monkeys than in rhesus monkeys. To examine virus shedding from monkeys, the presence and amount of virus in swabs was examined using RT-PCR and titration. The viral titer in nasal swabs increased in cynomolgus monkeys in the latter period of the experiment (Table 1). In two of the cynomolgus monkeys (#23 and #24), infectious virus was also detected in oral swabs. On the other hand, viral shedding from the mouth or nose of rhesus monkeys was detected in the early period of the experiment, but did not continue until the end of the experiment (Table 1). In addition, the difference in the amount of viral shedding in nasal swabs between species was significant (p=0.02, one-way repeated measures ANOVA). These results suggest that viral infection in the respiratory tract and viral shedding occurred more readily in the cynomolgus monkeys than in the rhesus monkeys, consistent with the higher and broader virus distribution in the cynomolgus monkeys.

**Table 1:** Viral shedding of HPAIV-infected macaques.

Swabs		Oral								Nasal							
Dpi		2		4		6		7		2		4		6		7	
Species	Sample	R	V	R	V	R	V	R	V	R	V	R	V	R	V	R	V
Cynomolgus	#23	-	NA	-	NA	+	ND	+	3.5	+	3.33	+	4	+	3.67	+	4.5
	#24	+	3.33	-	NA	-	NA	+	3	+	3	+	2.33	+	3.67	+	2.5
	#25	+	ND	-	NA	-	NA	-	-	+	ND	+	2	+	2.5	+	2.5
Rhesus	#29	+	ND	+	2.5	+	3.33	-	NA	+	ND	+	ND	+	ND	+	ND
	#30	+	<2	-	NA	-	NA	-	NA	+	3	-	NA	-	NA	-	NA
	#31	+	ND	-	NA	-	NA	-	NA	+	<2	-	NA	-	NA	-	NA

The presence of viral RNA in nasal or oral swabs of cynomolgus monkeys and rhesus monkeys was analyzed by RT-PCR (R). +: present; -: absent. The virus titer of viral RNA-positive samples was further analyzed. Data are presented as log_10_TCID_50_/ml. ND: not detected, NA: not analyzed.

 It has been reported that HPAIV causes systemic infection, but it depends on the strain of virus [16]. We examined the distribution of the virus in many organs to determine the potential of the strain to cause systemic infection, and also examined whether the two macaque species show a similar viral distribution. The heart and chest lymph nodes were positive in both species (Table 2). In several monkeys, viral RNA was also detected in other organs including the liver, spleen, jejunum and cerebellum using conventional PCR, suggesting that this strain is able to cause systemic infection, including infection of the central nervous system.

**Table 2:** HPAIV infection in extrapulmonary organs of cynomolgus and rhesus monkeys.

Species	ID	Heart	LN-c	Liver	Kidney	Spleen	LN-m	Bladder	Jejunum	Rectum	Brain stem	Cerebrum	Cerebellum
Cynomolgus	#23	+	+	+	-	-	-	-	-	-	-	-	-
	#24	-	+	+	-	-	-	-	+	-	-	-	+
	#25	+	+	-	-	-	-	-	-	-	-	-	-
Rhesus	#29	+	+	-	-	-	-	-	-	-	-	-	-
	#30	+	+	-	-	+	-	-	-	-	-	-	-
	#31	+	+	-	-	+	-	-	-	-	-	-	-

The presence of viral RNA was analyzed by RT-PCR. +: present; -: absent. LN-c: lymph node in the chest; LN-m: mesenteric lymph node.

**Figure 5:**
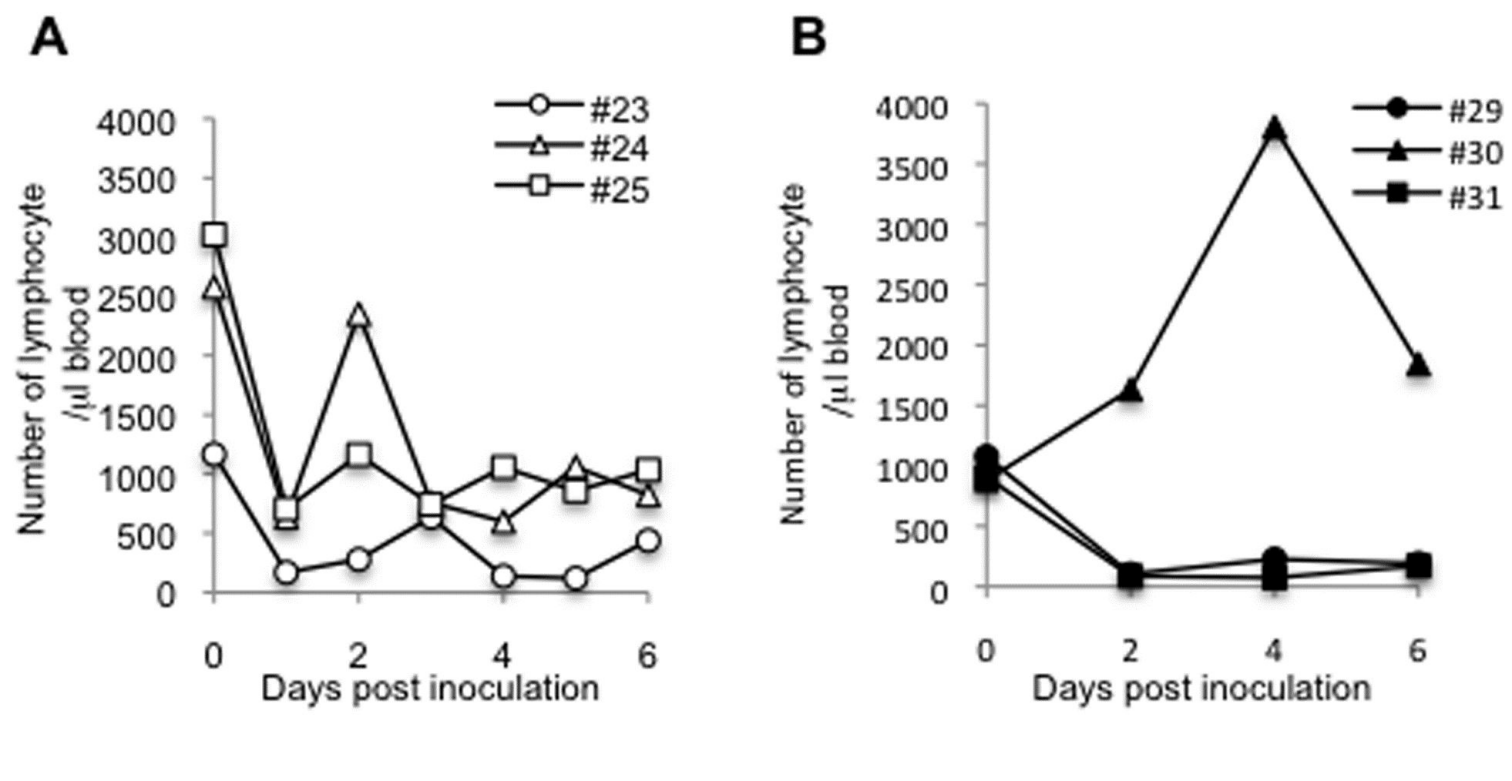
Numbers of leukocytes and lymphocytes in blood of HPAIV-infected macaques. Blood was collected daily from cynomolgus (A) or rhesus monkeys (B) at 0, 2, 4, 6, and 7 dpi from rhesus monkeys. The number of lymphocytes was calculated from the number of total leukocytes and the lymphocyte ratio.

 Hematological analysis revealed that lymphocytes were decreased in all cynomolgus monkeys (Figure 5A). Rhesus monkeys (#29, #31) also showed decreased lymphocytes (Figure 5B), except for one monkey (#30) in which the viral distribution was very limited (Figure 4), showing that this HPAIV strain causes lymphopenia in macaques. 

**Figure 6:**
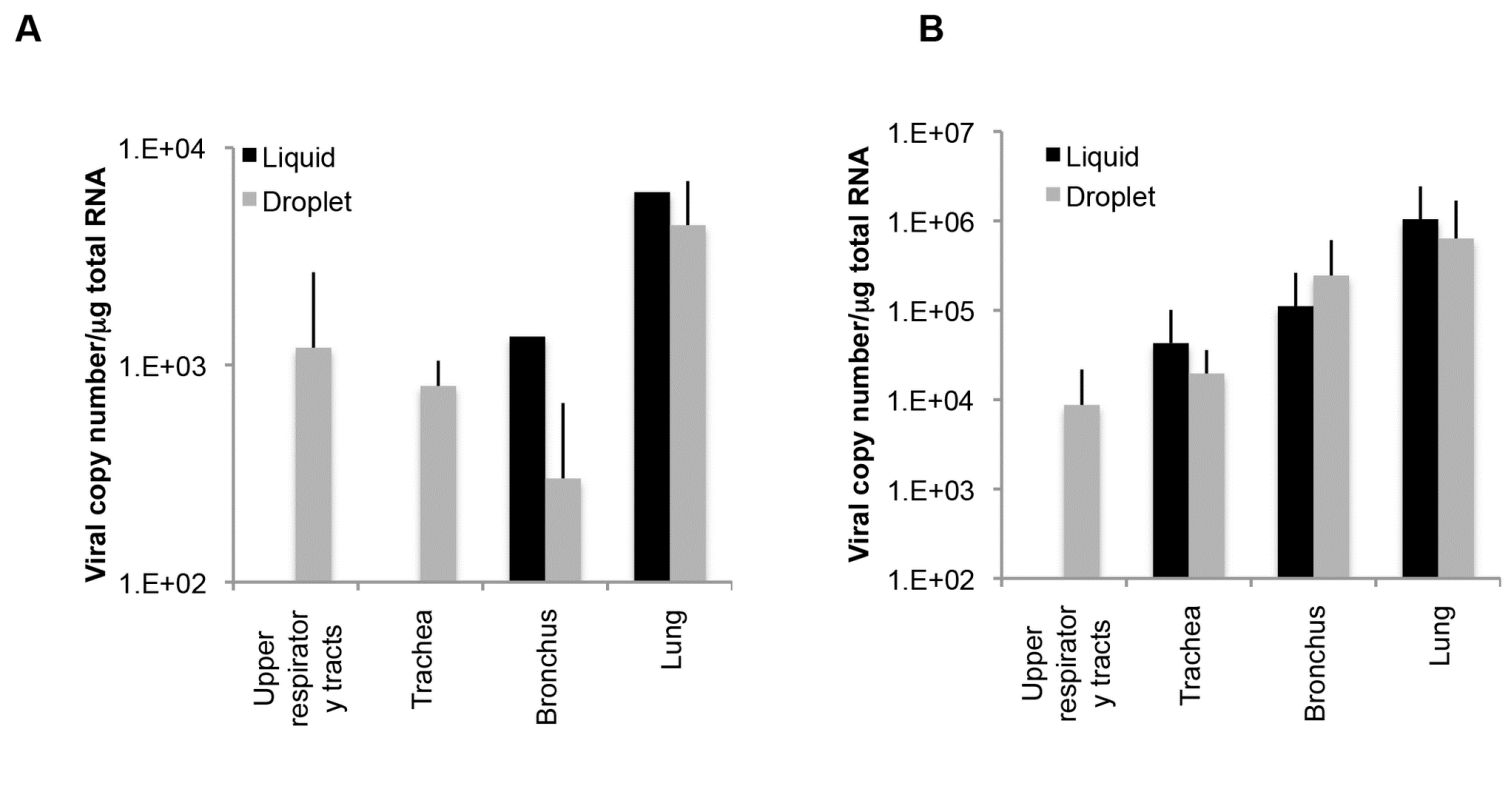
The amounts of viral RNA in the respiratory tract in rhesus and cynomolgus monkeys between different inoculation methods. (A) A rhesus monkey was inoculated with HPAIV via a catheter. Viral RNA in the respiratory tract (two parts for the upper respiratory tract, three parts for the trachea, two parts for the bronchus, and six parts for the lungs) was quantified by qRT-PCR. The total amount of viral RNA in each organ is indicated as the log value. Data on the three rhesus monkeys (#29−31) shown in Figure 4 was similarly analyzed and the mean±SEM for three monkeys is indicated. (B) Cynomolgus monkeys were inoculated with HPAIV via liquid exposure (n=2) or droplet exposure (n=1). Data were analyzed similarly to (A), including data for cynomolgus monkeys (#23−25). Data are means±SD was indicated.

 Previous inoculation routes to the trachea have involved infusion of a certain amount of virus solution using a catheter. The new method used in the current study was droplet exposure, which was suspected to be a more natural infection route. To compare the effects of these different inoculation routes, we inoculated an additional rhesus monkey by a previously reported method using a catheter. In monkey 26, viral RNA was detected in the bronchus and lungs but not in the upper respiratory tracts or the trachea (Figure 6A). On the other hand, other monkeys inoculated via droplet exposure (#29, #30, and #31) were positive for viral RNA in the upper respiratory tract and trachea, suggesting that the viral distribution in the respiratory organs was greater with droplet exposure than with the liquid inoculation method in this experiment, although the number of tested animals was too small to draw a definitive conclusion. To examine whether this broader viral distribution following inoculation via droplet exposure is also observed in cynomolgus monkeys, we inoculated two cynomolgus monkeys (#34 and #59) with the virus via liquid exposure and four cynomolgus monkeys (#23, #24, #25, and #44) via droplet exposure (Figure 6B). Viral RNA was not detected in the upper respiratory tracts of monkeys inoculated via catheter exposure, but was detected in those of monkeys inoculated via droplet exposure. Therefore, our results suggest that droplet exposure causes a broader distribution of HPAIV than does liquid inoculation. 

### Pathological progression in rhesus monkeys infected with higher infectious doses

**Figure 7:**
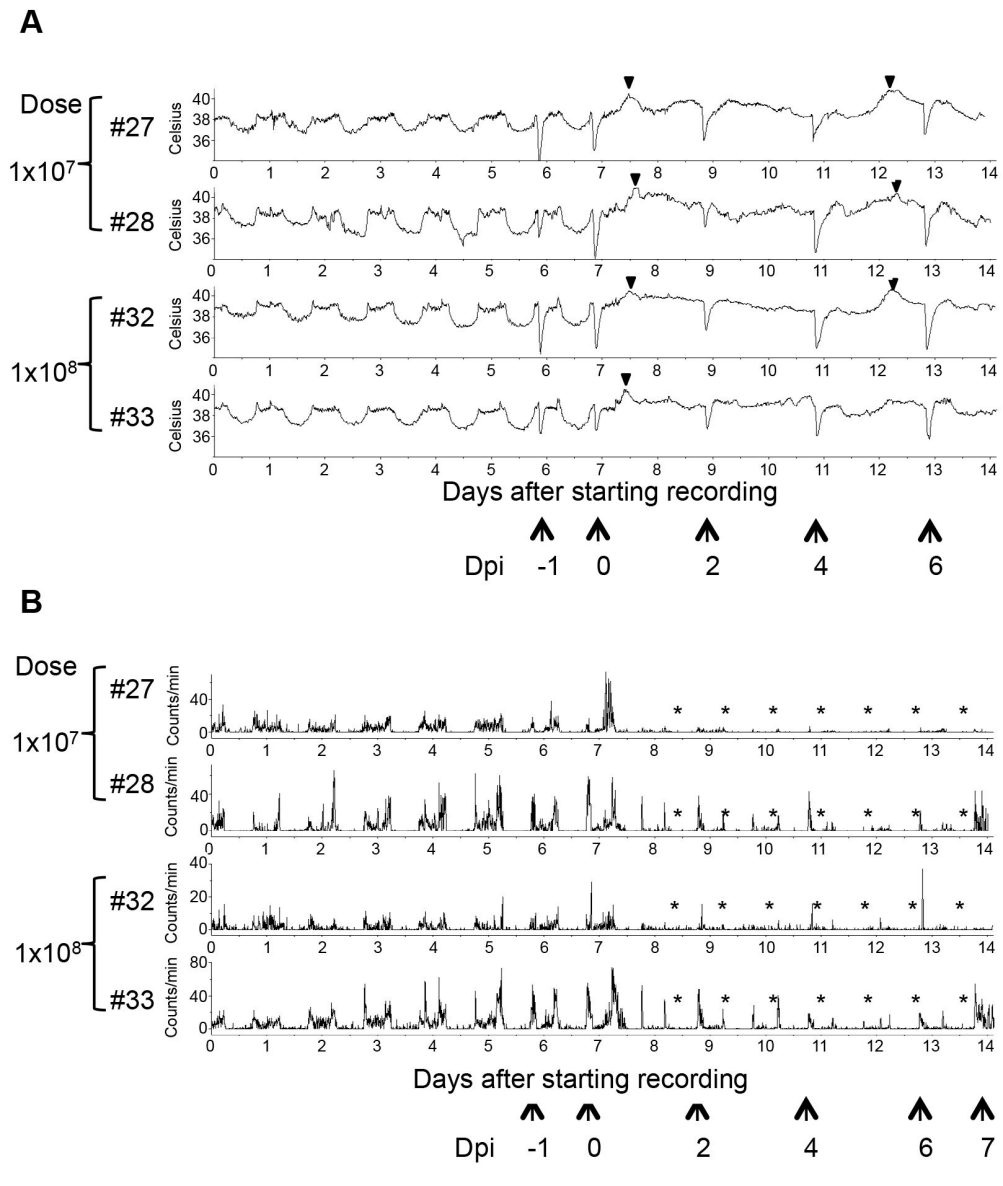
Body temperature and activity in rhesus monkeys challenged with higher infectious doses of HPAIV. Body temperature (A) and activity (B) of rhesus monkeys were monitored. The onset of fever was observed (arrowheads). *Period with decreased activity; AUC was decreased to ≤ 50% of the pre-inoculation level. The time points of anesthesia are indicated by arrows. Hyperthermia was caused by anesthesia.

 Cynomolgus monkeys are usually used as model animals for HPAIV infection. Our findings suggest that rhesus macaques also represent a good species for an HPAIV infection model. Therefore, we further examined whether rhesus monkeys inoculated with the virus show symptoms of influenza in a dose-dependent manner. We inoculated four monkeys with a higher titer of the virus (1 × 10^7^ PFU/monkey or 1 × 10^8^ PFU/monkey; two monkeys for each dose) via intratracheal droplet exposure, and the intranasal and oral routes. The earliest symptom observed was fever from the night of the inoculation day (Figure 7A). Once fever started, the circadian cycle of body temperature disappeared and the high body temperature continued during the night. Moreover, the body temperature gradually decreased at day 4 but increased again at 5 dpi. This biphasic fever was also observed in a cynomolgus monkey after HPAIV inoculation (data not shown). This type of biphasic fever is observed in cases of human influenza [17]. 

**Figure 8:**
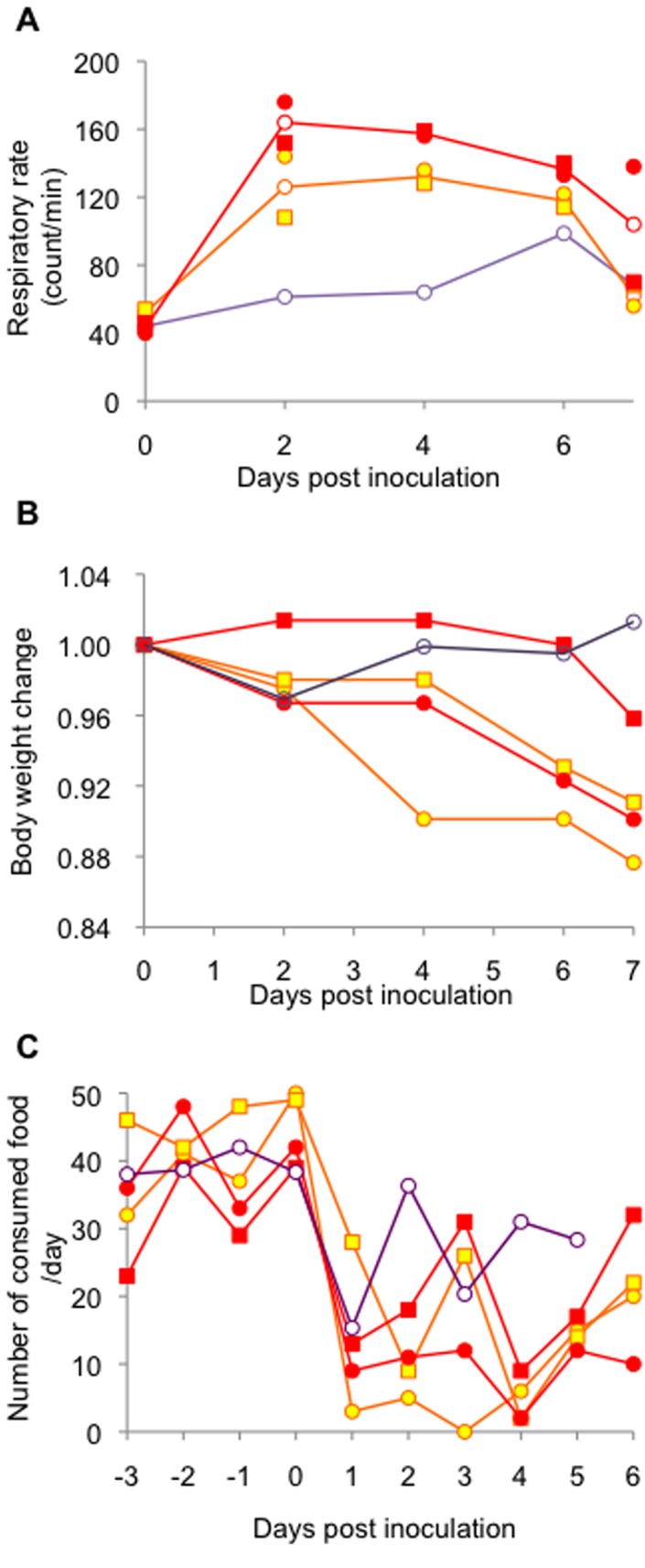
Clinical symptoms in rhesus monkeys challenged with HPAIV. Respiratory rate (A) and body weight (B) were measured soon after anesthesia. The amount of consumed food was recorded daily (C). Orange and red lines show the data of the monkeys inoculated with 1 × 10^7^ PFU (#27, circle; #28, square) or 1 × 10^8^ PFU (#32, circle; #33, square), respectively. A purple line shows the mean of the data of the monkeys inoculated with 3 × 10^6^ PFU.

 All of the challenged animals showed decreased activity from 1 dpi (Figure 7B). Each of the rhesus monkeys challenged with 1 × 10^7^ PFU (#27) or 1 × 10^8^ PFU (#32) showed apparent depression during the observational period. In addition, all of the monkeys inoculated with the higher infectious doses showed more severe or earlier tachypnea, reduced body weight and appetite loss (Figure 8). Cough and piloerection were also observed. These data suggested that higher infectious doses of the virus caused more severe clinical symptoms in rhesus monkeys. 

**Figure 9:**
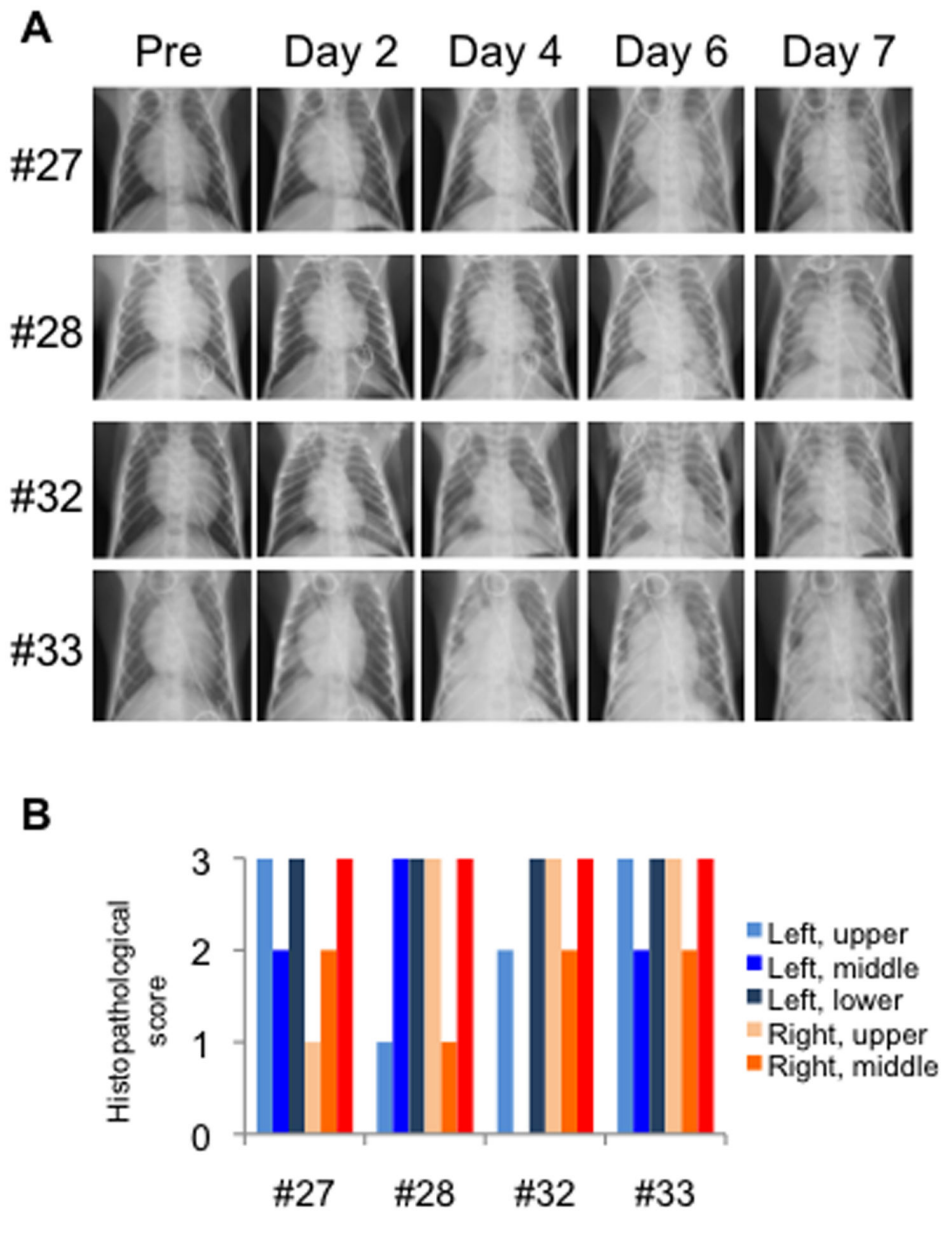
Progression of pneumonia in rhesus macaques after HPAI infection. (A) Radiographic photographs of the chest were taken every other day in rhesus macaques. (B). Histopathology of the lung lobes was scored as: 0, none; 1, mild; 2, severe. * Not analyzed.

**Figure 10:**
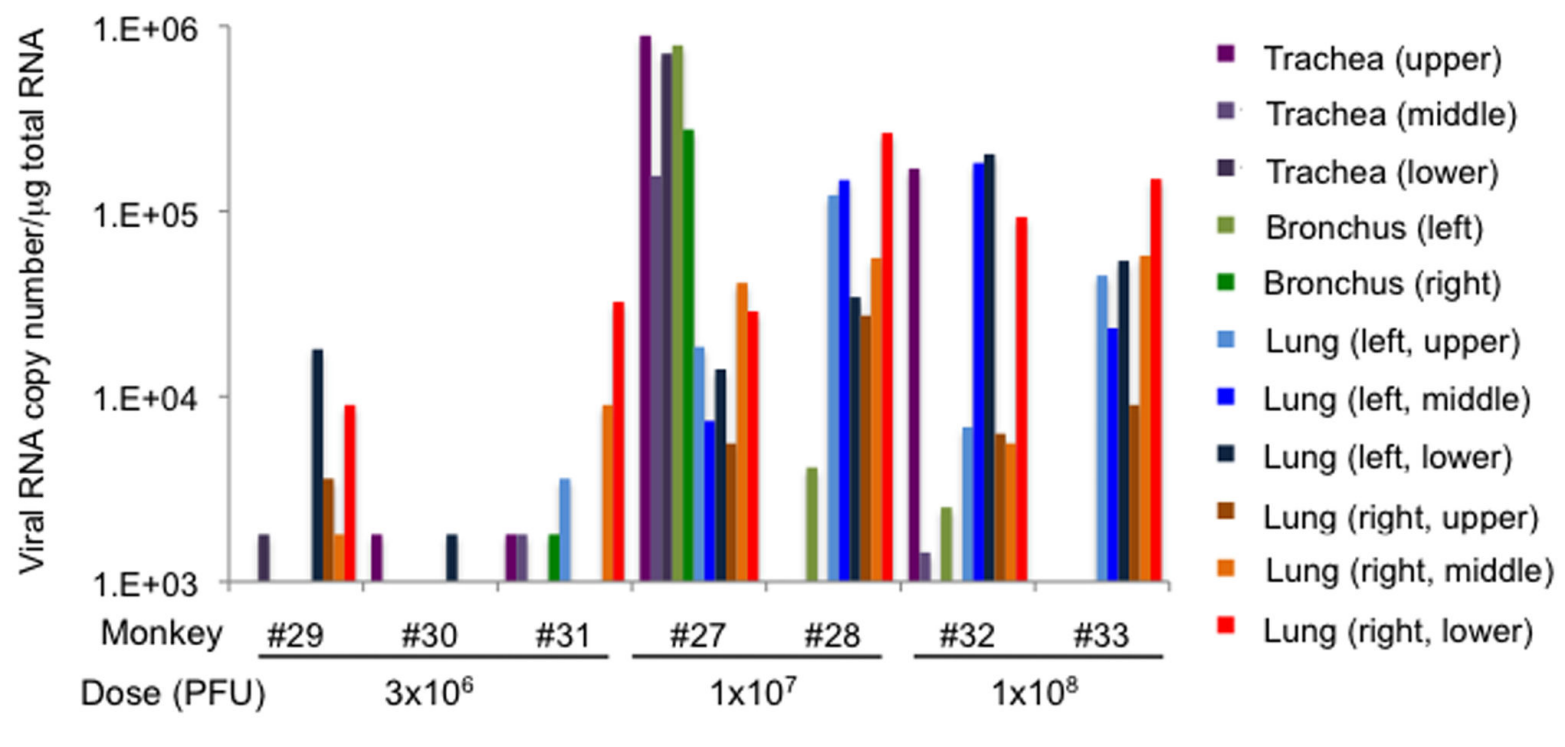
The amount of viral RNA in the respiratory tract among rhesus monkeys inoculated with different doses of HPAIV. Viral RNA was quantified by qRT-PCR. The log value of relative viral RNA content is indicated.

**Figure 11:**
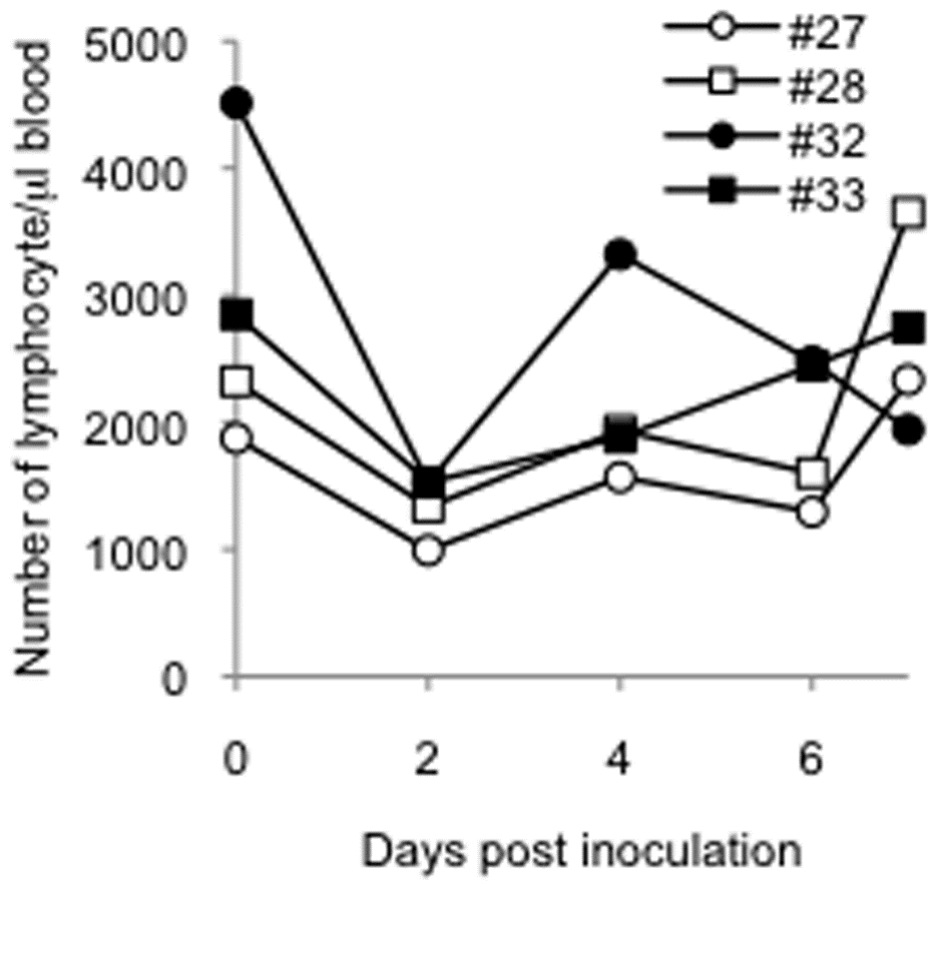
The numbers of lymphocytes after HPAIV infection. The number of lymphocyte was examined as described in Figure 5.

 Progression of respiratory disease was further examined by using radiographic imaging (X-ray) in rhesus monkeys (Figure 9A) under anesthesia. In rhesus monkeys challenged with 10^8^ PFU, signs of pneumonia were clearly observed from 2 dpi. Areas of pulmonary consolidation increased in distribution and density until 6 dpi. When rhesus monkeys were challenged with 10^7^ PFU, these signs were subtle at 2 dpi and became clearer at 4 dpi. All monkeys were euthanized at 7 dpi, and necropsy revealed that lesions in the lung occurred radially around the bronchus (data not shown). Histopathologic analysis showed broad lesions in the lungs (Figure 9B). These results suggested that droplet exposure with high infectious doses in rhesus monkeys was efficient at causing severe pneumonia. The distribution of the viral infection in the respiratory tract was broader (Figure 10, p<0.001, Chi Square Approximation) in monkeys challenged with higher infectious doses compared with that in monkeys challenged with 3 × 10^6^ PFU. The amount of viral RNA detected in the lungs increased with increasing infectious dose (Figure S2). The viral titers in swabs were under detectable levels (data not shown), which suggested that viral shedding hardly occurred in rhesus monkeys, even when the virus was growing in the lower respiratory tract. Viral distribution in extrapulmonary organs and a decrease in lymphocytes were observed, similar to the cases inoculated with 3 × 10^6^ PFU (Table 3, Figure 11). Notably, viral RNA was detected in the brains of both of the monkeys inoculated with 1 × 10^8^ PFU.

**Table 3:** HPAIV infection in extrapulmonary organs of rhesus monkeys challenged with higher infectious doses.

Dose (PFU)	ID	Heart	LN-c	Liver	Kidney	Spleen	LN-m	Bladder	Jejunum	Rectum	Brain stem	Cerebrum	Cerebellum	Spinal cord
1x10^7^	#27	+	+	+	-	+	-	-	+	-	-	-	-	-
	#28	+	+	-	+	+	-	-	+	-	-	-	-	-
1x10^8^	#32	+	+	-	-	+	+	-	-	-	-	-	+	-
	#33	+	+	-	-	+	-	-	-	-	-	-	+	+

The presence of viral RNA was analyzed by RT-PCR. +: present; -: absent. LN-c: lymph node in the chest; LN-m: mesenteric lymph node.

## Discussion

In this study, we found that a wild water bird-derived HPAIV strain (A/whooper swan/Hokkaido/1/2008) caused influenza onset in cynomolgus and rhesus monkeys similar to human cases of HPAIV infection with severe pneumonia. This finding indicates that this virus strain is pathogenic in primates. In addition, droplet exposure as an inoculation method is useful for analysis of pathogenesis in animal models, more closely mimicking natural infection. 

 Historically, macaque models of influenza virus infection have been studied since the 1940s using rhesus monkeys [18,19], but cynomolgus macaques have been used more often than rhesus macaques. The HPAIV infection model is not exceptional. We performed a detailed examination of HPAIV infection in rhesus monkeys in the present study. HPAIV was broadly distributed in rhesus as well as cynomolgus monkeys. In our study, the virus was detected not only in the respiratory organs but also in the extrapulmonary organs including the central nervous system (cerebellum and spinal cord), the lymphoid system (lymph node and spleen), and the digestive tract (jejunum) of rhesus monkeys. These findings suggest that the HPAIV strain used in this study causes systemic infection. Although another previous report using rhesus monkeys did not report systemic infection [4], a broader distribution of virus was unexpectedly observed in this study. In human cases, several studies have reported that HPAIV is distributed to the lymphoid system, the central nervous system and the digestive tract as well as the respiratory organs [20,21]. A cynomolgus monkey model showed a similar distribution [12]; thus, it is believed that cynomolgus monkeys present a better model for systemic infection. However, our results reveal that a rhesus monkey model is also a good model for studying systemic infection by HPAIV. 

 There are a few papers reporting the pattern of fever and the change in circadian rhythms in HPAIV-infected monkeys [11,12]. Cynomolgus monkeys infected with HPAIV (A/HongKong/156/97) via conventional inoculation showed a long fever period [12]. In another paper describing cynomolgus monkeys infected with HPAIV (A/Vietnam/1194/04) via conventional inoculation, the monkeys showed a shorter fever period [11]. In both experiments, the animals’ body temperature decreased at nighttime and, thus, circadian rhythms were maintained. In our study, the monkeys showed biphasic fever over a long period and their circadian rhythms disappeared, similar to the pattern in human cases. In addition, the lymphopenia observed in this study is consistent with one of the characteristic symptoms of HPAIV infection in humans [22-24]. Furthermore, we first demonstrated progression of pneumonia in monkeys by radiography until 7 days after HPAIV infection, consistent with human cases that show abnormalities on chest radiographs within a week of the onset of symptoms [25]. Thus, inoculation of monkeys with the strain of HPAIV used in this study provides a good model for studying the mechanisms underlying clinical symptoms.

 Considering virus inoculation route, the droplet exposure used in this study could cause broad virus distribution in respiratory organs, which might have resulted in respiratory symptoms such as tachypnea and pneumonia, indicating that the primary infection occurred in the respiratory tracts. Although our inoculation method included pouring of liquid into the mouth, which does not occur during actual virus transmission, it seems not to be responsible for the virus infection in the respiratory tracts, because liquid sample inoculated into the mouth should not flow into the trachea under normal conditions, in which the epiglottis is closed. In addition, the method is much closer to actual virus transmission than previous inoculation methods using pouring of liquid into the trachea, which is quite different from actual virus transmission. It was reported that H5N1 virus infection was not detected in bronchus of a rhesus macaque following intranasal inoculation [4]. In another paper, when HPAIV was inoculated via intratracheal infusion of the virus solution using a catheter, the virus was detected in respiratory organs including the trachea in three out of four monkeys at 3 dpi, but no virus was detected in any respiratory organs at 15 dpi [5]. Although virus replication was not examined at 7 dpi in the report, the virus might have been eliminated or remain with quite a low titer because it was described that the monkeys recovered on 4 and 5 dpi. In our present study, the virus was detected in the trachea of all monkeys, including the upper trachea. Broader distribution in respiratory tracts was also observed in cynomolgus monkeys. Therefore, it is suggested that droplet exposure was useful for causing respiratory infection with HPAIV.

 Because there are few reports on HPAIV infection in rhesus monkeys, the similarities and differences in HPAIV pathogenicity among primate species are not well understood. The results of our study suggested that HPAIV infected both rhesus and cynomolgus monkeys, and thus, both species are good models for HPAIV infection. In our study, there appeared to be several differences in pathogenicity between the two species. The distribution of the virus in respiratory organs was broader in the cynomolgus monkeys than in the rhesus monkeys. Consistently, viral shedding from cynomolgus monkeys was obvious, but it was hardly observed in the rhesus monkeys, while their lower respiratory tracts were well infected. In addition, the virus was distributed to extrapulmonary organs in both species, but distribution to the spleen was only observed in rhesus monkeys, suggesting that the rhesus monkey model would be useful for studying systemic infection with HPAIV. There might be a difference in anti-viral activity against influenza between the two macaque species, as it has been suggested that the anti-viral activity of TRIM5α against human immunodeficiency virus type 2 infection is different between rhesus and cynomolgus macaques [26]. It is also possible that the differences resulted from the fact that the rhesus monkeys we used in this study were younger than the cynomolgus monkeys. Whether the differences in susceptibility resulted from difference in species or the age of monkeys remains to be studied in the future. 

 In summary, the current study demonstrated that the A/whooper swan/Hokkaido/1/2008 strain isolated from a dead wild water bird, which is lethal to chickens [6], is pathogenic to primates, while HPAIV strains that are usually used for studies of pathogenicity in animal models are isolates from human patients. This finding suggests that caution should be paid to any contact with wild birds. Our data also showed that the monkey model generated by droplet inoculation as a new method with this strain of HPAIV is useful for the investigation of the pathogenicity of respiratory and systemic infection with HPAIV in humans, and for studies on the efficacies of new vaccines or therapies. 

## Supporting Information

Figure S1 **Comparison of inoculation methods.** BALB/c mice (12 to 14-week-old) were administered 50 μl of 0.1% methylene blue using a MicroSprayer (PennCentury, IA-IC-M) to the trachea (A−C) or using a micropipette to the nostril (D−F). The lung was collected soon after the administration and observed from dorsal (A, D), and ventral (B, E) sides of the lung, or inside of each lobe (C, F). Methylene blue was observed more widely in the lungs of mice administered with a MicroSprayer than in the lungs of those administered with a micropipette.
(TIF)

Figure S2 **Dose dependency of the detected viral RNA in the lungs.** Average viral RNA copy number in the lung lobes of each monkey was plotted with HPAIV infectious doses. Correlation coefficient was shown in the graph.
(TIF)

Table S1 **Summary of inoculation method.**
(TIF)
