# Supplementary figures and images for: Experimental Infection of Macaques with a Wild Water Bird-Derived Highly Pathogenic Avian Influenza Virus (H5N1)
Source: PLoS One. 2013 Dec 18;8(12):e83551. doi: 10.1371/journal.pone.0083551 (PMC3867452; doi:10.1371/journal.pone.0083551)

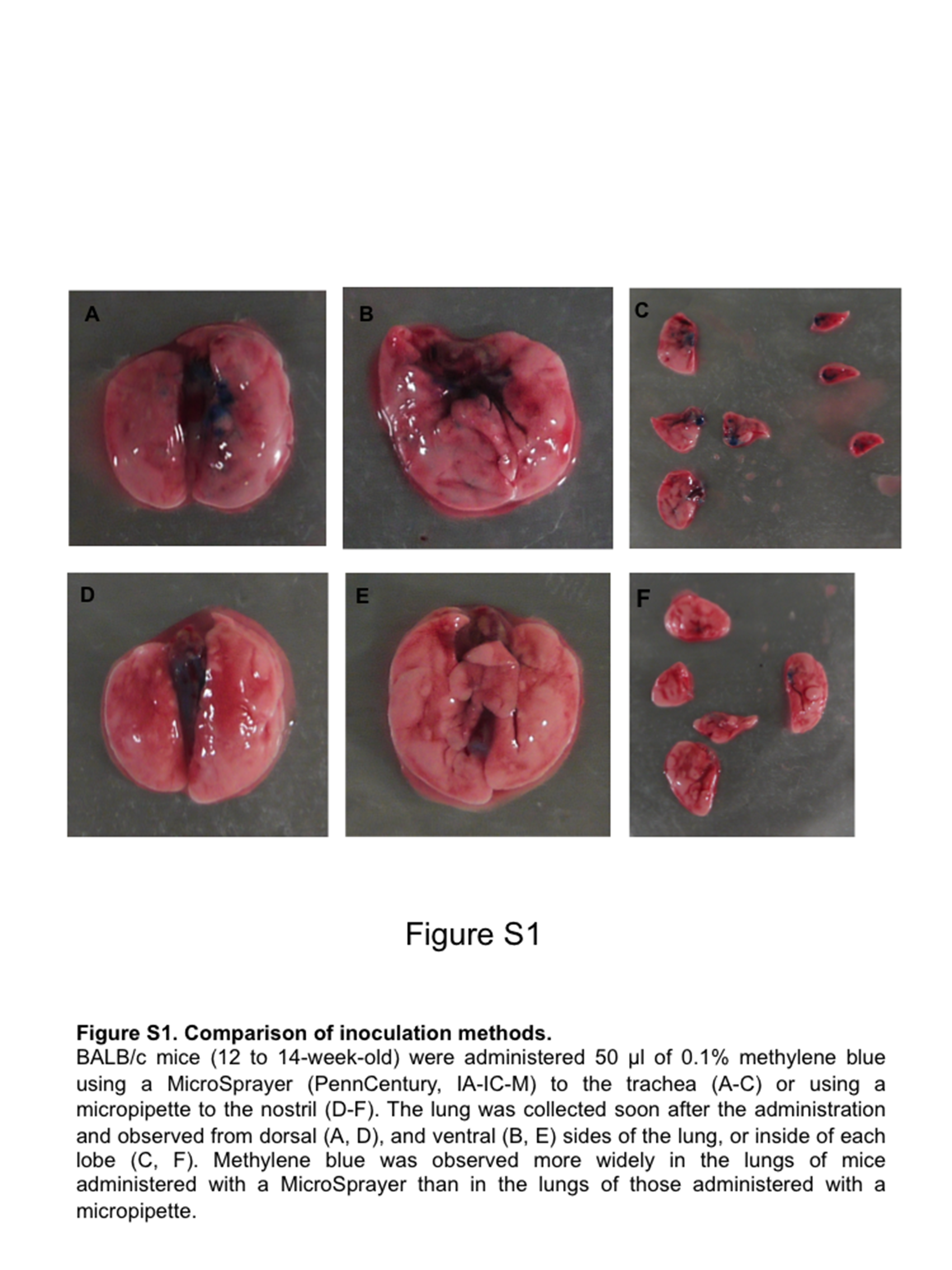

Supplement: Figure S1 — Comparison of inoculation methods. BALB/c mice (12 to 14-week-old) were administered 50 μl of 0.1% methylene blue using a MicroSprayer (PennCentury, IA-IC-M) to the trachea (A−C) or using a micropipette to the nostril (D−F). The lung was collected soon after the administration and observed from dorsal (A, D), and ventral (B, E) sides of the lung, or inside of each lobe (C, F). Methylene blue was observed more widely in the lungs of mice administered with a MicroSprayer than in the lungs of those administered with a micropipette. (TIF) [file pone.0083551.s001.tif]

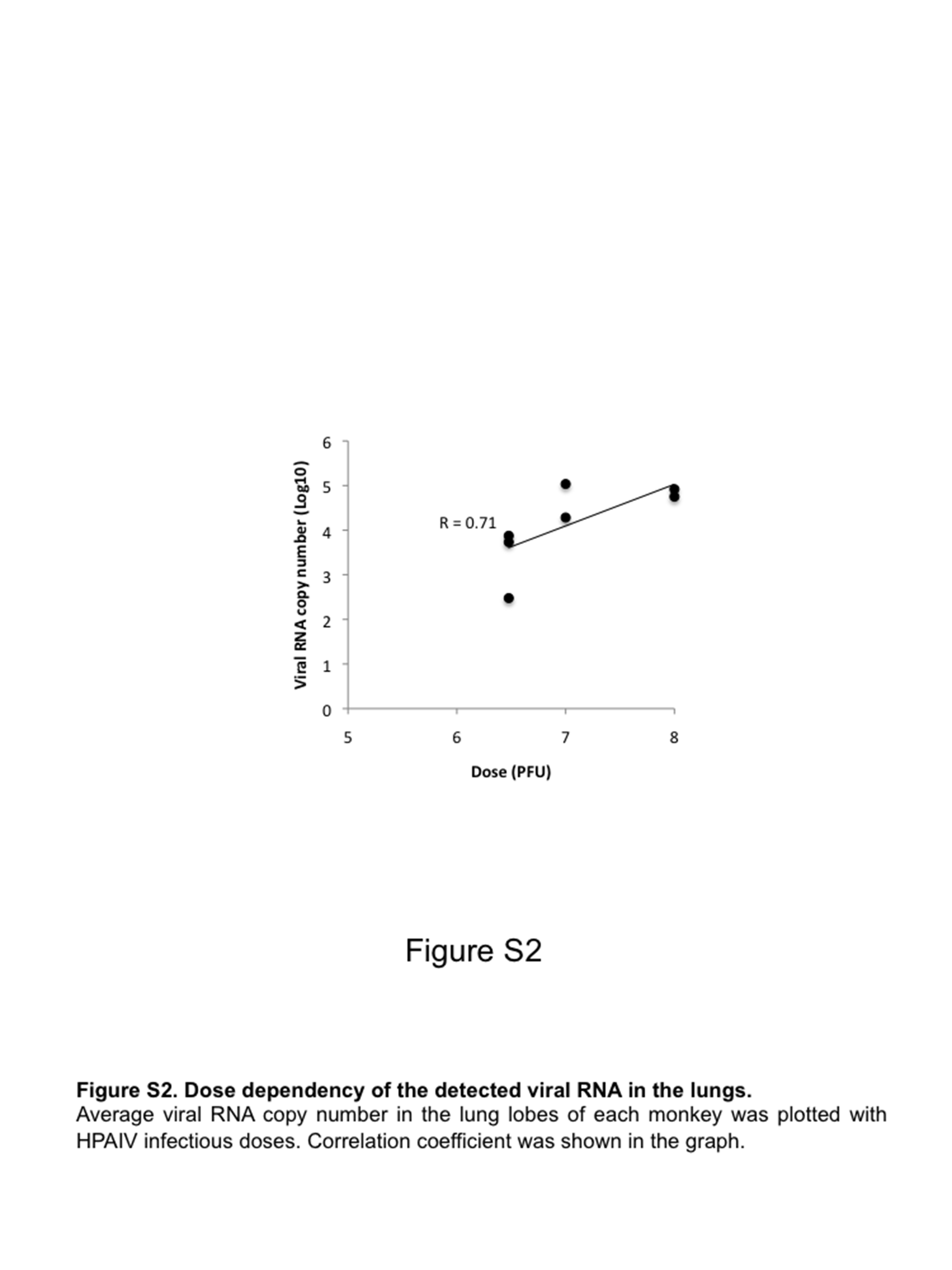

Supplement: Figure S2 — Dose dependency of the detected viral RNA in the lungs. Average viral RNA copy number in the lung lobes of each monkey was plotted with HPAIV infectious doses. Correlation coefficient was shown in the graph. (TIF) [file pone.0083551.s002.tif]
